# The implication of liquid biopsies to predict chemoresistance in pancreatic cancer

**DOI:** 10.20517/cdr.2021.01

**Published:** 2021-04-14

**Authors:** Elisabetta Pietri, Rita Balsano, Matilde Coriano, Fabio Gelsomino, Francesco Leonardi, Simona Bui, Letizia Gnetti, Raffaele Dalla Valle, Ingrid Garajová

**Affiliations:** ^1^Medical Oncology Unit, University Hospital of Parma, Parma 43126, Italy.; ^2^Department of Oncology and Hematology, University Hospital of Modena, Modena 41124, Italy.; ^3^Unit of Pathological Anatomy, University Hospital of Parma, Parma 43126, Italy.; ^4^Department of Medicine and Surgery, University of Parma, Parma 43126, Italy.; ^#^Authors contributed equally.

**Keywords:** Pancreatic cancer, liquid biopsy, CTC, ctDNA, exosomes

## Abstract

Pancreatic cancer is one of the most aggressive diseases among solid tumors. Most patients are diagnosed with advanced or metastatic disease and are characterized by poor chemosensitivity. Therefore, earlier diagnosis and novel therapeutic possibilities for pancreatic cancer patients are urgently needed. Liquid biopsy is an emerging technology that allows the noninvasive sampling of tumor material. Nowadays, liquid biopsy has shown promising results as diagnostic, prognostic and predictive biomarkers, but it has not yet been universally adopted into regular use by clinicians. In this review, we describe different components of liquid biopsy, especially circulating tumor cells, circulating tumor DNA and exosomes and their potential clinical utility for pancreatic cancer patients.

## INTRODUCTION

The most common pancreatic neoplasm is pancreatic ductal adenocarcinoma (PDAC), occurring in more than 85% of pancreatic tumor cases^[1]^. It is one of the most lethal types of human cancer with 5-year survival rate less than 10%^[2-7]^. Radical surgical resection remains the only potential curative option for PDAC patients; however, the probability of relapse is high. The average survival of resected patients ranges from 12 to 20 months, and the 5-year survival rate after surgery and adjuvant systemic therapy is approximately 20%^[5-7]^. Around half of PDAC patients are diagnosed with distant metastasis. Unfortunately, progress in the management advanced-stage disease has been very modest in the last decades. Actually, chemotherapy regimens (gemcitabine and abraxane or FOLFIRINOX) are gold standard in the treatment of locally advanced or metastatic PDAC, although providing only slight improvements in OS, reaching at best few months^[8-10]^. No target therapies and no immunotherapy approaches are nowadays clearly effective in PDAC. Only the implementation of genetic testing can change a very narrow treatment landscape for small subsets of patients with actionable aberrations: in particular, in *BRCA1/2 *mutated setting, olaparib (PARP inhibitor) might be proposed as maintenance strategy^[11]^. Therefore, only few different treatment combinations are currently available to metastatic PDAC patients, and it is important to switch between them wisely.

**Figure 1:**
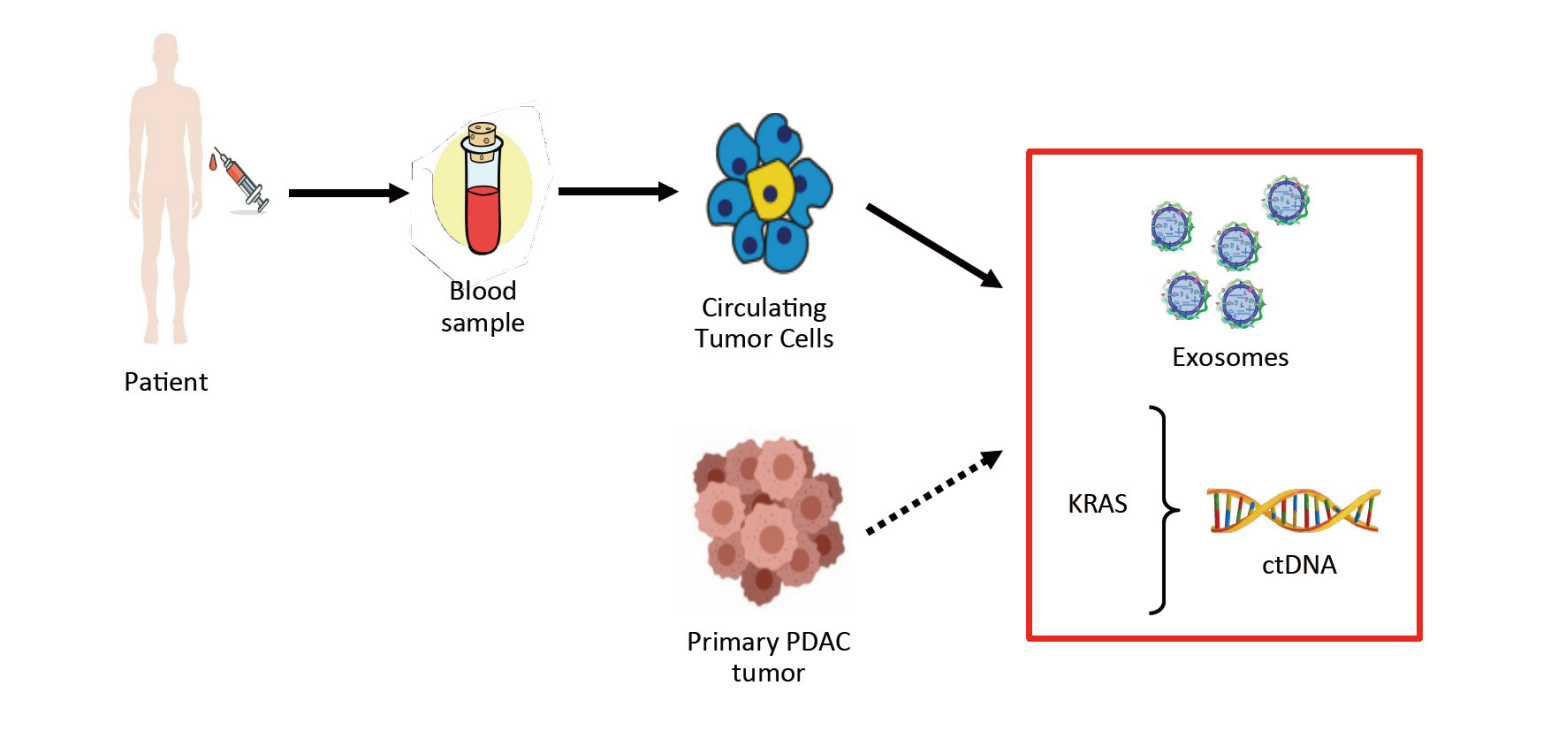
Detection of CTCs, exosomes and ctDNA by collecting blood samples from PDAC patients. PDAC: Pancreatic ductal adenocarcinoma; ctDNA: circulating tumor DNA.

**Figure 2:**
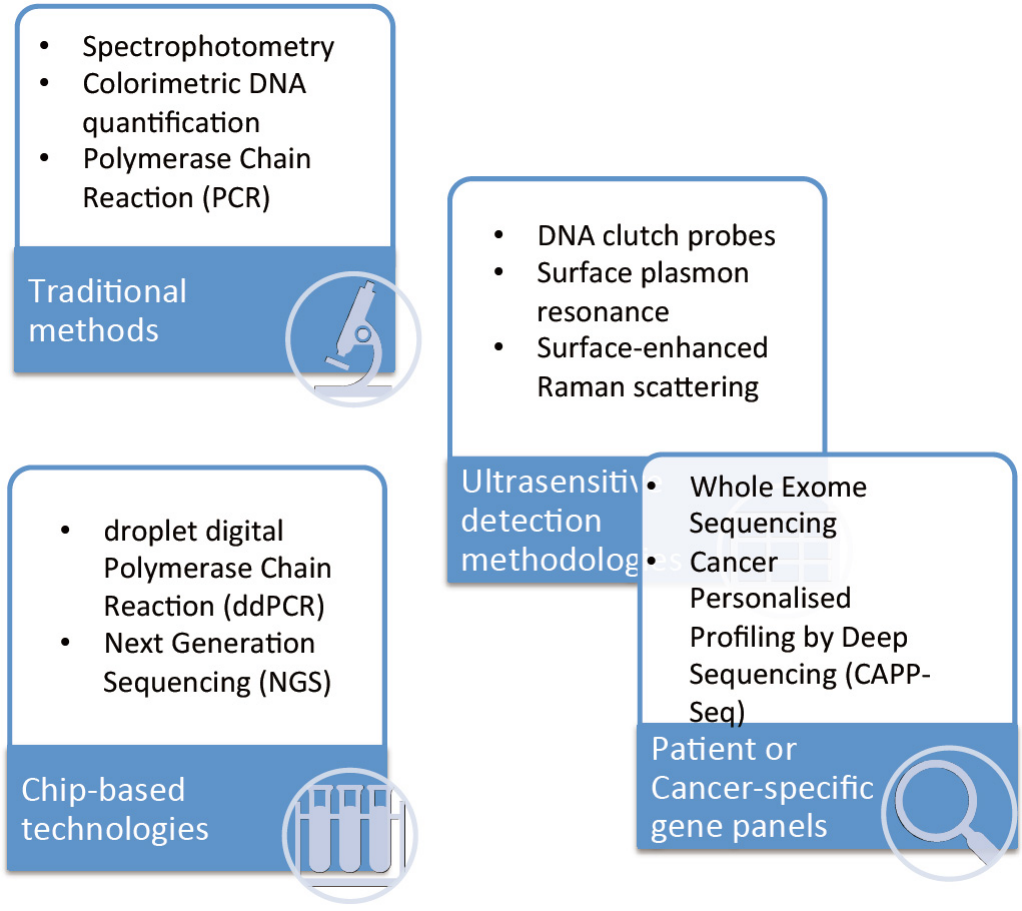
Different approaches for detection of DNA aberrations on liquid biopsy^[12,93]^.

Pancreatic adenocarcinomas release their components into circulation through either the exocrine or endocrine system, and, for this reason, blood, pancreatic juice, stools, saliva and urine contain these biological markers and can be a source for their detection and analysis^[12]^. Blood is undoubtedly the most common fluid employed as source of tumor components, and “liquid biopsy” is a novel technology that allows the noninvasive sampling of tumor material. In particular, tumor components released in body fluids such as circulating tumor cells (CTCs), circulating tumor DNA (ctDNA), vesicles and exosome (EVs) have been studied as predictors of chemoresistance in PDAC [Figure 1]. One of the potential clinical application of liquid biopsies is to identify specific tumor molecular characteristics that could improve the diagnostic process, have prognostic or predictive significance and might be used to determine the potential chemosensitivity or chemoresistance in PDAC patients. Different approaches for detection of DNA aberrations on liquid biopsy are depicted in Figure 2.

## MOLECULAR PATHOLOGY OF PANCREATIC CANCER

In this section, we describe molecular classification of PDAC in order to understand genetic alterations that, together with tumor microenvironment, influence pancreatic tumor cell behavior.

**Figure 3:**
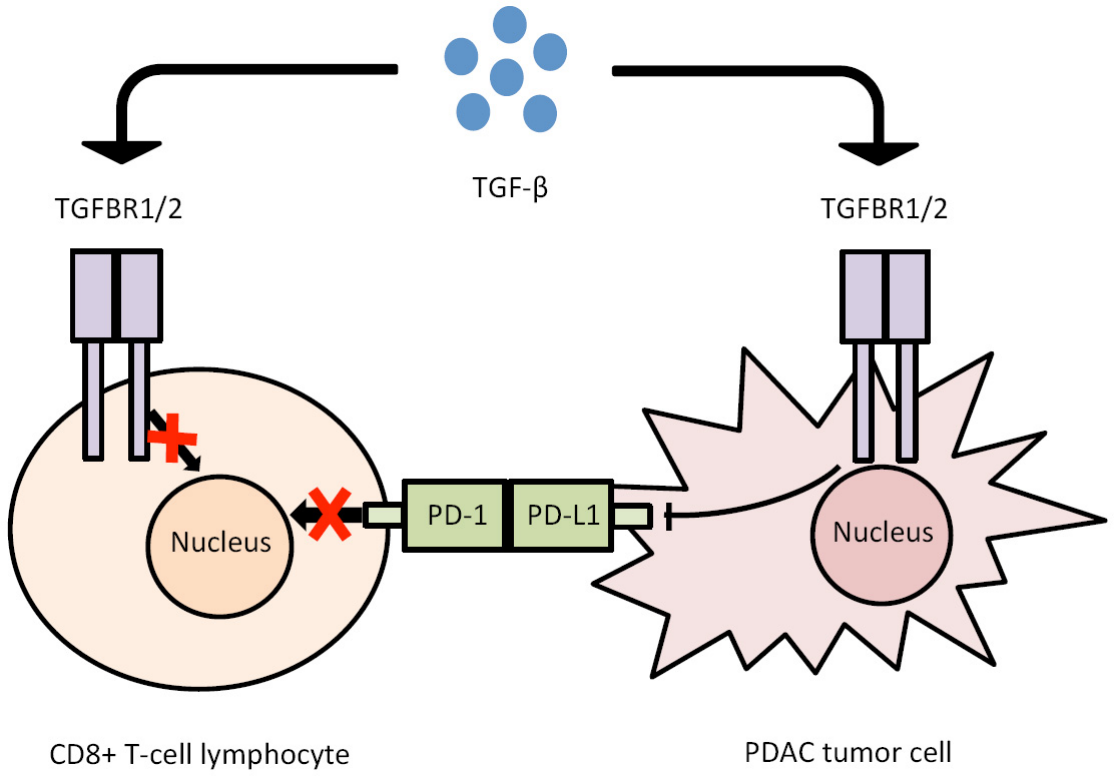
TGF-β upregulates PD-L1 expression and inactivates CD8+ cytotoxic lymphocytes, thus resulting in a reduced immune-response to PDAC. PDAC: Pancreatic ductal adenocarcinoma; TGF-β: transforming growth factor-β.

Genetic alterations are acquired (or somatic) in most pancreatic cancers. However, in 10%-20% of PDAC the are hereditary (or germline) mutations, prominently regarding DNA mismatch repair (MMR) genes such as *ATM*, *BRCA1*, *BRCA2* and *PALB2*^[13]^. PDAC is a heterogeneous molecular disease, showing a high level of inter-tumor genetic heterogeneity^[14]^. The intra-tumor heterogeneity is caused by unequal distribution of cancer cell subclones in the same tumor (spatial heterogeneity). Moreover, the tumor changes over the time: different genetic alterations are selected during tumor development (temporal heterogeneity)^[15]^. Studies analyzing genomic alterations in resected PDACs found near 2000 gene mutations, the four most commonly mutated being *KRAS* (90%), *CDK2NA *(90%), *TP53* (75%-90%) and *SMAD4* (50%)^[16,17] ^[Table 1 and Figure 3]. Raphael *et al*.^[18]^ performed a study with an integrated genomic, transcriptomic and proteomic characterization of 150 PDAC specimens. They confirmed the prevalence of *KRAS* mutations in 93% of PDAC samples. In the remaining 7% of PDAC samples, *KRAS *gene mutations were not identified. However, these *KRAS* wild tumors harbored molecular alterations in other oncogenic drivers. In addition, this integrated transcriptomic and proteomic profiling revealed RNA and protein subtypes that indicate clinically significant subsets of disease^[18]^. Analyzing signaling pathways involved in the process of PDAC tumorigenesis and progression, *KRAS* has emerged as dominant, but other multiple additional pathways are genetically altered including cell cycle progress, DNA damage control, *TGF-β*, Hedgehog and WNT/Notch^[19-21]^.

**Table 1:** The most frequent gene mutations and respective pathways involved in PDAC carcinogenesis, according to Bailey *et al*.^[17]^

**Mutation**	**Frequency**	**Pathways**	**Ref.**
*KRAS*	92%	*MAPK*	[17]
*G12D/G12V*	82%	*PI3K*	[17]
*G13*	14%		[17]
*Q61*	< 1%		[17]
*CDK2NA/TP53*	78%	*G1/S *checkpoint	[17]
*KDM6A/SETD2/MLL*	24%	Histone modification	[17]
*SMAD/TGFBR*	47%	*TGFβ*	[17]
*ARID1A/PBRM1/SMARCA4*	14%	*SWI/SNF*	[17]
*BRCA1-2/ATM/PALB2*	12%	*BRCA*	[17]
*RNF43*	5%	*WNT*	[17]

The global gene expression profiling molecular approach has allowed identifying different PDAC expression subtypes. There are three principal classifications: Colisson with “classical”, “quasi-mesenchymal” and “exocrine”-like; Moffit with “normal” and “activated” stroma, “classical” and “basal-like” tumor subtypes; and Bailey with “squamous”, “pancreatic progenitor”, “immunogenic” and “ADEX”^[17,22,23]^. Different PDAC transcriptional subtypes show prognostic differences. Analyzing the three classifications, we can conclude that Collisson’s quasi-mesenchymal subtype has the worst survival, particularly compared to classical tumor subtype. The best prognosis is presented by the combination of classical tumor subtype and Moffitt’s normal stroma subtypes, in comparison to the association of basal-like subtype with activated stroma subtypes. Moreover, Bailey’s squamous subtype has a worse outcome than the other three subtypes^[14]^. In 2015, Waddell *et al*.^[24]^ identified four different PDAC subtypes based on a number of structural variation events contained in tumors: (1) a stable subtype with tumor genomes containing ≤ 50 structural variation events located randomly through the genome; (2) a locally rearranged subtype exhibiting intra-chromosomal rearrangements clustered on one or few chromosomes; (3) a scattered subtype containing 50-200 structural rearrangements scattered through the genome; and (4) an unstable subtype with > 200 structural rearrangements scattered through the genome. Most unstable tumors are those with *BRCA* signature associated with mutations of *BRCA1/2* and *PALBB2*. Patients with *BRCA* mutational signature (a putative surrogate measure of deficiencies in DNA repair) had better response to platinum-based therapy. Recently, Sinkala *et al*.^[25]^ used algorithms to identify sets of proteins, mRNAs, miRNAs and DNA methylation patterns, in order to identify biomarkers to differentiate pancreatic cancers subtypes. This approach can identify mRNA, protein or miRNA biomarkers and data regarding their concentrations can be matched with drug responses from either cancer patients or cell lines to predict drug responses of PDAC^[25]^.

To understand intrinsic characteristics of pancreatic cancer, it is necessary to analyze composition of tumor microenvironment (TME) that deeply interacts with cancer cells determining growth, invasion and metastasis of each neoplasm. Even these interactions are molecularly determined. Histologically, PDAC can be described as a “hypertrophic scar with sparse neoplastic cells” because it consists of cancer cells sparse among stromal cells which represent up to 85% of tumor mass. Stromal cells include different cell components, in particular pancreatic stellate cells (PSCs), regulatory T cells (Tregs), myeloid-derived suppressor cells (MDSCs) and tumor-associated macrophages (TAMs). These stromal cells secrete extracellular components [e.g., extracellular matrix (ECM), matrix metalloproteinase (MMP), growth factors and transforming growth factor-β (TGFβ)]. All together, these components represent TME^[19]^. TME has two major characteristics: dense desmoplasia and extensive immunosuppression. Desmoplasia is an extensive fibrosis at the primary site that can establish a hypoxic microenvironment, which induces invasion, proliferation, inhibition of apoptosis and metabolic changes^[26]^. Pancreatic cancer cells are able to induce immunosuppression by inhibiting CD8^+^ T cells from playing an important role in killing tumor cells and upregulate the exiting regulatory immune cells^[27]^. Moreover, TME can induce angiogenesis for tumor blood supply, new lymphatic vessels growth that are vehicles for cancer cells spread and the process of epithelial-mesenchymal transition (EMT) by which cancer cells lose their cell-cell adhesion capacity with subsequent invasion through the basement membrane creating pre-metastatic niche formation, where circulating tumor cells can promote PDAC progression or induce tumor dormancy^[28]^.

In conclusion, today, a universally accepted subtyping of pancreatic tumors (based on their mutations, expression transcription or protein profiles) has not been followed by success in increased effectiveness of available therapies or identifying tumor biomarkers for early diagnosis and therapeutic response monitoring in PDAC patients^[29]^.

## CIRCULATING TUMOR CELLS AS PREDICTORS OF CHEMORESISTANCE IN PANCREATIC CANCER

CTCs are tumor cells which enter the bloodstream by breaching the basement membrane of the pancreatic acinus and ducts, stroma and vessel walls. This transition is driven by two principal mechanisms: forces of interaction created internally to the tumor mass and the EMT program^[30]^. On the one side, some epithelial tumor cells undergo passive intravasation, escaping from the primary tumor^[31]^. On the other side, some tumor cells undergo EMT, secrete digestive enzymes, lose their polarity and cell-cell/matrix adhesion and gain migratory properties^[32]^. There are two models of CTCs intravasation: single CTCs detachment from tumor site or collective CTCs invasion^[33]^. This means that CTCs may travel as single cells or clusters^[34,35]^. CTCs which access the intravascular space have to overcome selection processes exerted by the hemodynamic stress of bloodstream and programmed cell death (termed “anoikis”) occurring when cells detach from the surrounding extracellular matrix^[36]^ and immune cell attacks^[12,30]^. There is evidence that malignant cells possess specific characteristics that enable them to survive mechanical stresses. These properties allow CTCs to survive during their passage through the circulation^[37]^. Moreover, CTCs develop anoikis resistance by the EMT process, promoting proteins linked to anoikis resistance and cell-remodeling, thus modifying energy metabolism, growth factor receptors expression, membrane microdomains, reactive oxygen species and lipid rafts^[38]^. In the bloodstream, CTCs must evade immune system after leaving the immune privileged site of the primary tumor. CTCs have developed strategies to escape immune response, such as intracellular transcriptional changes leading to the upregulation of PD-L1 inhibiting T cell-mediated immunity^[39]^; production of inhibitor cytokines that negatively modulate NK cell anti-tumor activity^[40]^; EMT-related processes promoting epithelial cell plasticity leading to less immunogenicity^[41]^; and promotion of platelets number and activity that modulate NK activity, reduce CTCs shear stress in the bloodstream and increase CTCs extravasation^[42]^. CTCs traveling in the bloodstream are not a monolithic entity, but a heterogeneous one. The CTCs population’s heterogeneity is induced by acquired genetic and epigenetic alterations, EMT events, clustering phenomenon and microenvironment-associated cues^[43]^. There is evidence of epithelial CTCs expressing epithelial markers such as epithelial cell adhesion molecule (EpCAM); mesenchymal-like CTCs expressing traditional mesenchymal markers such as vimentin; CTCs co-expressing epithelial and mesenchymal markers; and CTCs expressing positive stem cells (CSC) markers such as CD4 and CD133 [which are called circulating tumor stem cells (CTSCs)]^[44]^. EMT promotes acquisition of mesenchymal and CSC markers^[45]^, inducing so-called EMT-CTCs. CTSCs and mesenchymal-like CTCs, derived from the EMT event, have higher invasion and migratory potentials. CTSCs demonstrate self-renewal and differentiation into multiple cell types, capacities that make them more capable of reaching and colonizing distant organs^[46]^. Further, CTSCs and mesenchymal-like CTCs contribute to promote resistance to conventional anticancer treatments by many mechanisms such as increasing drug efflux, promoting cell dormancy and increasing genomic DNA repair^[33]^. A fraction of CTCs, called disseminated tumor cells (DTCs), has the capacity of extravasation, entering distant sites (seeding process) and then interacting with new a microenvironment and progressing toward metastases^[47]^. DTCs undergo mesenchymal-to-epithelial transition (MET) with subsequent proliferation and dissemination, or, alternatively, they might enter in dormancy and generate metastasis a long time after their establishment in the distant site^[48]^.

CTCs in pancreatic cancer are rare, around one CTC per 10^8^ hematologic cells per mL of blood or even less in early-stage cancer^[49]^. In pancreatic tumors, CTCs entering the bloodstream are conducted to the liver through the portal vein. Thus, thoss in the portal vein might provide a better representation of the CTC population^[50]^.

In pancreatic cancer, CTC detection was identified as a possible diagnostic biomarker and their enumeration correlates with staging, prognosis and tumor resectability^[51]^. A meta-analysis analyzing 623 PDAC patients demonstrated that the patients with positive CTCs had poorer progression free survival (PFS) and overall survival (OS) than the group of PDAC patients without CTCs. This might suggest that CTCs could be a promising diagnostic and prognostic biomarker in PDAC patients^[52]^. In another study, Ren *et al*.^[53]^ showed the presence of CTCs in 80.5% of stage III and IV PDAC patients before any therapy. Examining the CTCs at different time-points (prior to initiation of 5-fluorouracil-based chemotherapy and after seven days of treatment), the presence of CTCs decreased to 29.3%, suggesting a potential role of CTCs in early response to anticancer prediction^[53]^. Another recently published systematic review and meta-analysis of 19 studies assessing 1320 PDAC patients showed that CTC positive patients had significantly shorter OS and PFS than CTC-negative patients^[54]^. This again suggests that CTCs may have a prognostic role in PDAC patients.

Referring to chemoresistance, in prostate and breast cancer, characterization of CTCs have shown capacity of early prediction of treatment response^[55,56]^. In small-cell lung cancer (SCLC), genomic analysis of CTCs with relative molecular classifier has allowed assigning patients into chemosensitive or chemorefractory group^[57]^. The possible prediction of therapy response based on CTCs is currently under evaluation in different cancer types, such as prostate, breast, colorectal, SCLC and gastric cancer, in both preclinical and animal models^[58]^. To our knowledge, the only study that evaluated CTCs as predictor biomarker of treatment response in PDAC was published by Yu *et al*.^[59] ^The authors analyzed gene expression profile of CTCs in 50 patients with metastatic or locally advanced PDAC^[59]^. They created and validated Pharmacogenomic (PGx) modeling of tumor tissue to predict efficacy of chemotherapeutic agents in preclinical cancer models. CTCs isolated from patients’ blood were profiled and the PGx model was used to predict effective and ineffective chemotherapeutic regimens. The authors concluded that, using previously created PGx models to predict chemotherapy efficacy, the clinical benefit was seen for PDAC patients treated with chemotherapy regimens predicted to be effective *vs.* chemotherapy regimens predicted to be ineffective with regard to PFS and OS.

The confirmation that CTCs might function as biomarkers of chemoresponse is essential to identify “non-responders” and therefore to avoid ineffective treatments^[60]^. Generally, CTCs represent blood available cells originating from primary tumor or metastatic lesions giving phenotypic and molecular tumor characterization in real time. Therefore, they might have importance in tumor diagnosis, staging and prognosis, as well as prediction of response to cancer treatment.

### CTDNA AS CHEMORESISTANCE PREDICTORS IN PANCREATIC CANCER

Both benign and malignant cells undergo physiological processes such as apoptosis or necrosis with subsequent release of genetic material into circulation which might be detected peripherally as cell-free DNA (cfDNA)^[61]^. The portion of cfDNA which refers specifically to the tumor-derived portion is called circulating tumor DNA (ctDNA)^[61]^. Many studies have found a correlation between tumor aggressiveness and the quantity of the absolute amount of ctDNA^[62]^. As PDAC exhibits the highest frequency of *KRAS* mutations, *KRAS* is likely to be the best-characterized tumor-related gene that also occurs at an early stage of PDAC^[61]^. Therefore, several studies have focused on ^mut^*KRAS *ctDNA detection (in particular, G12V, G12D and G12R mutations) and its potential role in PDAC development, extrapolating prognostic and predictive data. Kruger *et al*.^[63]^ found *KRAS* mutated ctDNA in 75% of pancreatic tumors, slightly higher (79%) if only metastatic patients were considered. They also noticed that the quantity of ^mut^*KRAS *ctDNA significantly differed between patients with non-progressive disease and progressive disease at time of first re-staging. They observed an early increase in ^mut^*KRAS *ctDNA during the first two days after chemotherapy followed by a decrease in ^mut^*KRAS *ctDNA, usually after the first or second week of chemotherapy. This trend was seen above all in PDAC patients with good response or disease stabilization at the time of radiological re-staging. An initial increase of ^mut^*KRAS *ctDNA is observed in most patients, although an increase after two weeks might correlate with disease progression. Therefore, the authors suggested Day 14 might be a crucial time point to evaluate early kinetics^[63]^. Hadano *et al*.^[61]^ evaluated 105 patients who underwent surgery for resectable PDAC and identified *KRAS* mutation in both tumor and plasma samples before resection in 33 patients. *KRAS *status concordance between tumor specimens and matched plasma samples was 100%. Moreover, the presence of ctDNA, and in particular ^mut^*KRAS *ctDNA, in plasma samples was significantly associated with poorer outcome in both disease-free survival (DFS) (6.1 months *vs.* 16.1 months) and OS (13.6 month *vs.* 27.6 months) analyses. This study suggests a potential prognostic role of ctDNA at early stage PDAC: patients with high ctDNA levels at diagnosis may benefit from neoadjuvant chemotherapy before undergoing surgery. The changes in cDNA levels in pre-surgery and post-surgery liquid biopsy might provide a proof of therapeutic efficacy and management strategies of PDAC^[61]^. Wei *et al*.^[64]^ explored application of cfDNA and ctDNA profiling in monitoring tumor burden change following FOLFIRINOX treatment in 38 PDAC patients. Their results show a high degree of concordance between radiologic therapy response and dynamics of ctDNA allele fraction^[64]^. Cheng *et al*.^[65]^ collected plasma samples from 13 metastatic PDAC patients. They observed that the presence of ^mut^*KRAS *ctDNA correlated with radiologic tumor responses^[65]^. Watanabe *et al*.^[66]^ analyzed the importance of one-point determination of ^mut^*KRAS *ctDNA levels before chemotherapy or surgery treatments, in order to predict treatment outcome. They did not prove any association between the presence of ^mut^*KRAS *ctDNA before surgery and relapse-free survival. Nevertheless, the presence of ^mut^*KRAS *ctDNA before chemotherapy start was correlated with worse prognosis (the median OS of PDAC patients with and without detection of ^mut^*KRAS *ctDNA was 15.8 and 33.7 months, respectively). Furthermore, the emergence of ^mut^*KRAS *ctDNA in PDAC patients was associated with poor prognosis. The emergence of ^mut^*KRAS *ctDNA within six months of chemotherapy termination correlated with worse PFS. ^mut^*KRAS *ctDNA in patients disappeared in response to drug treatment^[66]^. Similar results were presented by Del Re *et al*.^[67]^, who noticed that PDAC patients undergoing chemotherapy treatment and displaying ^mut^*KRAS *ctDNA increase on the 15th day of therapy had disease progression; conversely, the early ^mut^*KRAS *ctDNA modulation was not associated with tumor response. Therefore, ^mut^*KRAS *ctDNA is a potential biomarker of chemosensitivity/chemoresistance to anticancer treatment in PDAC patients. Monitoring the levels or the emergence of tumor molecular alterations in ^mut^*KRAS *ctDNA during anticancer treatment is a potential biomarker to monitor treatment resistance or response^[67]^.

## EXOSOMES AS PREDICTORS CHEMORESISTANCE IN PANCREATIC CANCER

Exosomes are extracellular vesicles that contain proteins, microRNAs (miRNAs) or messenger RNA (mRNA). They act as an important mediator of intercellular communication. They can regulate or even modify their surrounding microenvironment. The components of exosomes can therefore serve as cancer biomarkers. It is possible to detect exosomes by isolating them from various cell fluids including blood^[68]^, serum^[69]^, saliva^[70]^. In comparison to ctDNA, exosomes have a longer circulating half-life. As tumor cells produce exosomes without pause, their detection in peripheral blood is independent of certain events such as apoptosis or cell necrosis^[71-73]^. The components of exosomes were studied as diagnostic, prognostic and prognostic biomarkers in PDAC, as well as for novel therapeutic approaches. In one study, expression of exosomes containing miRNA-483-3p was higher in PDAC patients compared with patients with non-malignant IPMN^[74]^. Goto *et al*.^[75] ^studied serum miRNAs enclosed in exosomes in PDAC patients and patients with IPMN. They concluded that in particular three miRNAs (miR-191, miR-21 and miR-451a) showed elevated expression levels in patients with PDAC and IPMN and therefore might be used as biomarkers for early PDAC detection^[75]^. Further, expression of exosomal miR-191/21/451 was significantly elevated in patients with PDAC and IPMN compared to healthy controls^[75]^. In another study, the plasma levels of exosomes containing miR-196a and miR-1246 levels were significantly higher in PDAC patients as compared to the healthy population^[76]^. Su *et al*.^[77]^ identified five miRNAs (miR-16-2-3p, miR-890, miR-3201, miR-602 and miR-877) that have diagnostic potential of early PDAC diagnosis. Further, Zhu *et al*.^[78]^ showed how different levels of microRNA expression might distinguish patients with PDAC and healthy volunteers, appearing to be diagnostic markers. The potential use of miRNA as predictors of response to chemotherapy has been widely studied in PDAC. For example, miR-21 increases cell proliferation and the expression of factors involved in metastasis formation^[79]^. The reduction of miR-21 levels has been shown to be predictive of response to gemcitabine therapy^[80]^, while the reduction of miR-17-5p expression is predictive of response to nab-paclitaxel in PDAC patients^[81]^. Miyamae *et al*.^[82]^ showed that upregulation of plasma miR-744 contributed to worse PFS of non-resectable PDAC patients who underwent gemcitabine-based chemotherapy and therefore might be a useful tool to monitor chemoresistance in PDAC. In addition, the reduced tissue expression of MiR-10b represents a potential marker of response to neoadjuvant chemotherapy in PDAC patients^[83]^. In another study, it has been shown that reduction of miR-181a-5p plasma levels correlated with response to therapy with FOLFIRINOX. Moreover, the reduction was associated with better PFS and OS^[84]^. Interestingly, it has been shown that chemotherapy can induce the increase of not previously present miRNAs. In the study of Xia *et al*.^[85]^, chemotherapy with gemcitabine caused an increased expression of miR-155, a hypothesized acquired chemoresistance biomarker. In addition, several studies showed the diagnostic potential of microRNAs in PDAC. Unfortunately, even if numerous studies have been published, microRNAs have not been confirmed as universal biomarkers in PDAC patients. The use of different samples and studies involving different populations, as well as the attempt to use experimental elements, has been a limitation. The potential solution to this issue could be to carry out studies on as homogeneous as possible populations with defined tools, in order to reach a conclusive value.

Recently, the cell surface proteoglycan glypican-1 (GPC1) on tumor exosomes was identified. GPC1+ circulating exosomes (crExos) were analyzed in the serum of PDAC patients. The authors demonstrated that GPC1+ crExos were able to distinguish between healthy subjects and patients with a benign pancreas disease from patients with early- and late-stage PDAC^[86]^. Moreover, levels of GPC1+ crExos correlated with outcome in PDAC patients after radical surgery. The authors concluded that GPC1+ crExos might be considered as a diagnostic biomarker to detect early PDAC stages. However, the role of GPC1+ crExos is controversial. Frampton *et al*.^[87] ^adopted ELISA to quantify GPC1 levels in PDAC tissues and crExos; they concluded that GPC1+ crExos levels may correlate with tumor burden and response to surgical resection, but they are not useful to distinguish between benign and malignant PDAC lesions preoperatively or to rate the aggressiveness of the tumor. The authors of the two cited papers used different techniques: Melo *et al*.^[86]^ adopted techniques such as anti-GPC1 antibody labeled beads and subsequently flow cytometry, while Frampton *et al*.^[87]^ used ELISA, which is more reproducible and clinically amenable in a standard hospital laboratory. Interestingly, Buscail *et al*.^[88]^ combined quantification of GPC1+ crExos with CTC detection in order to increase their sensitivity and negative predictive value. They observed a negative predictive value of 100% and an overall diagnostic accuracy of 91%. This result could be useful in early stages of PDAC when the tumor is likely releasing fewer circulating biomarkers such as CTCs and exosomes. It could be crucial to make more rapid and effective decisions about the treatment in the early setting of such an aggressive disease^[88]^.

Some authors have provided evidence on the possible use of exosomes for therapeutic approaches. Aspe *et al*.^[89]^ used exosomes for surviving delivery to pancreatic cancer cell line (MiaPaCa-2) with subsequent restoration of gemcitabine sensitivity in pancreatic cancer cell. Bernard *et al*.^[90]^ investigated PDAC patients with potentially resectable tumors. They demonstrated that an exosomal DNA *KRAS *mutant allele fraction (MAF) peak of 1% was associated with PDAC progression, almost two months earlier than CT scan progression. Moreover, in borderline resectable PDAC patients who underwent systemic therapy with neoadjuvant intent, the exosomes DNA *KRAS* MAF kinetics before and after the completion of therapy correlated with disease progression and therefore an indication for no further surgical resection^[90]^. All these findings make exosomes a sensitive tool for early PDAC diagnosis, as well as possible prognostic and predictive biomarkers.

Liquid biopsy offers the possibility to analyze other cancer biomarkers: serum proteases comprising matrix metalloproteinase (MMPs) 1, 3 and 9; urokinase plasminogen activator (UpA); cathepsin-B and -E; and arginase. These proteases are over- or under-expressed in solid tumors and have a role in malignant progression (including tumor angiogenesis, invasion and metastasis) and immune dysregulation in cancer. These proteases take part in a proteolysis signal network of proteolysis interaction which in turn interacts with other signal networks such as chemokines, cytokines and kinases co-opted to promote tumor progression^[91]^. Arginase, cathepsin-B and -E, MMP-1 and -3 and UpA have been established as suitable serum markers of PDAC patients. Therefore, analysis of this panel of enzymes by means of a liquid biopsy is promising for early PDAC detection^[92,93]^.

## FUTURE DIRECTIONS AND CONCLUSIONS

Nowadays, pancreatic cancer is among the deadliest solid tumors. There is also a lack of personalized therapeutic approaches because pancreatic cancer biopsies are often inadequate for molecular characterization^[62]^. In this review, we give an overview of the current literature on CTCs, ctDNA and exosomes in pancreatic cancer (see also Table 2). Unfortunately, in a clinical context, no liquid biopsy is actually used in pancreatic cancer, in any setting. This is due to the lack of standardization of detection methods, as well as insufficient sensitivity and specificity of the possible biomarkers. Liquid biopsy has the potential to be incorporated into different phases of PDAC patients care. It would definitely be helpful as a diagnostic tool because the pancreas is hardly accessible to tissue biopsy due to its retroperitoneal position. Another valuable area in which liquid biopsy can be utilized is as a guide for resectable tumors in order to decide between immediate surgery or neoadjuvant treatment. In particular, detection of ctDNA, typically by the presence of mutated *KRAS*, might be a signal of microscopic metastases that could spare the futile major surgery. Another field where liquid biopsy might be helpful is their utility for monitoring response to different treatments. In conclusion, the implementation of liquid biopsies in clinical context represents a new hope for precision medicine and personalized treatments for PDAC patients.

**Table 2:** The prognostic performance of different circulating markers in different studies

**Study**	**Type of study**	**Number of patients**	**Outcome**	**Findings**
Wang *et al*.^[54]^	Systemic review and meta-analysis	1320	OS and PFS	Shorter OS and PFS in CTC positive PDAC pts
Meijer *et al*.^[84]^	Biological prospective study	54	OS and PFS	miR-18a-5p declined levels are correlated with improved OS and PFS
Abue *et al*.^[74]^	Biological prospective study	32	Compare miR-483-3p, miR-21 plasma samples of PDAC, IPMN and healthy control	miR-483-3p discrimines PDAC from IPMN; miR-21 associated with potential metastatic
Miyamae *et al*.^[82]^	Biological prospective study	94	Compare miR-615-5p, -744, -575, -557, -675, and -550a plasma samples of PDAC and healthy control	miR-744 is related to poor PFS
Preis *et al*.^[83]^	Biological prospective study	155	Evaluate the expression of miR-10b, miR-21, miR-155, miR-196a and miR-210 in PDAC samples	miR-10b associated with a improved response to neoadjuvant therapy
Bernard *et al*.^[90]^	Biological prospective study	425	Clinical utility of ctDNA and exoDNA	*KRAS* mutant allele fraction (MAF) marker of progression; the exoDNA *KRAS* MAF related to progression
Kruger *et al*.^[63]^	Exploratory study	83	Response prediction of mut*KRAS *ctDNA	mut*KRAS *ctDNA predictive of early response and therapy surveillance
Hadano *et al*.^[61]^	Exploratory study	105	OS	Shorter OS in ctDNA+ PDAC patients
Watanabe *et al*.^[66]^	Exploratory study	78	RFS, PFS, OS	mut*KRAS *ctDNA predictive of prognosis and therapeutic responses; mut*KRAS *ctDNA not associated with recurrence or prognosis if detected before surgery or chemotherapy
Del Re *et al*.^[67]^	Exploratory study	27	OS, DCR	mut*KRAS *ctDNA potential biomarker of chemosensitivity/chemoresistance

OS: Overall survival; PFS: progression free survival; CTC: circulating tumor cell; PDAC: pancreatic ductal adenocarcinoma; ctDNA: circulating tumor DNA.

## DECLARATIONS

### Copyright

© The Author(s) 2021.
